# A Rare Cause of Scrotal Swelling: Transitional Cell Carcinoma of the Bladder Presenting as a Testicular Metastasis

**DOI:** 10.1155/2011/284121

**Published:** 2011-04-06

**Authors:** W. Mahmalji, S. Jain, M. Stower

**Affiliations:** ^1^Department of Urology, York District Hospital, Wigginton Road, York YO31 8HE, UK; ^2^Department of Urology, St James, University Hospital, Beckett Street, Leeds LS9 7TF, UK

## Abstract

A 72-year-old Caucasian male who presented with haematuria in July of 2000 was found to have a large left-sided bladder tumour. He underwent a transurethral resection of the tumour and surveillance program. In October 2008 he underwent a transurethral resection of the prostate (TURP). Histology of the prostatic chippings showed poorly differentiated TCC with prostatic invasion. A CT of his chest abdomen and pelvis revealed no lymph node involvement or metastatic spread. He therefore underwent a cystoprostato-urethrectomy with ileal conduit formation, in December 2008. In May 2010 the decision was made to perform a left inguinal orchidectomy as he presented with a craggy mass of his left testis, and there were clinical concerns that this was a tumour. Histology revealed that the left testis had been wholly replaced by a tumour. Taking into account his previous urological history, the features of this tumour are consistent with metastatic TCC, which is very rare.

## 1. Case Report

A 72-year-old Caucasian male who presented with haematuria in July of 2000 was found to have a large left-sided bladder tumour, and urine cytology revealed malignant cells. A renal ultrasound scan (USS) at the time was normal. Transurethral resection of the tumour (TURBT) showed a G2 pT1 transitional cell carcinoma (TCC) of the bladder. A check cystoscopy in 2002 revealed a red patch over the previous resection site; and biopsies revealed transitional cell carcinoma in situ (Tis) which was managed by a six-week course of intravesical BCG. He continued cystoscopic surveillance, unfortunately in March 2005 further cystoscopic biopsies again revealed transitional cell carcinoma in situ (Tis), this was managed by further instillations of intravesical BCG. He again underwent a surveillance cystoscopy programme with biopsies when indicated, which were unremarkable until June 2008, where biopsies showed a reoccurrence of Tis again managed by six-week course of intravesical BCG.

In October 2008 he underwent a transurethral resection of the prostate (TURP) as he had presented with acute retention of urine. Histology of the prostatic chippings showed poorly differentiated TCC with prostatic invasion. A CT of his chest abdomen and pelvis revealed no lymph node involvement or metastatic spread. He therefore underwent a cystoprostato-urethrectomy with ileal conduit formation, in December 2008. Surveillance CT scans (including urogram phases) in June and November 2009 were unremarkable.

**Figure 1:**
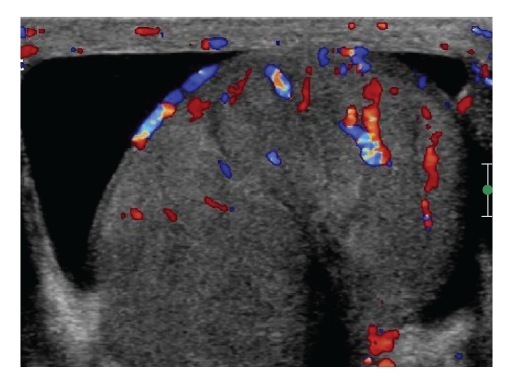
A scrotal USS revealed an enlarged testis with hypervascularity compatible with orchitis, May 2010.

He presented to the urology department in January 2010 complaining of a 2-week history of left testicular pain. Examination was unremarkable, but a scrotal USS suggested epididymo-orchitis as a potential diagnosis; thus, a 2-week course of ciprofloxacin (500 mg twice daily) was prescribed and his symptoms improved. However his pain never really settled but he did not represent until May 2010. On this occasion examination revealed a craggy mass and generalised scrotal swelling. A scrotal USS revealed an enlarged testis with hypervascularity compatible with orchitis (Figure 1).

 The decision was made to perform a left inguinal orchidectomy to clinical concerns that this was a tumour. Histology revealed that the left testis had been wholly replaced by a tumour composed of islands of pleomorphic cells with surrounding desmoplastic stroma and the ghost outlines of atrophic seminiferous tubules. Immunohistochemical studies showed the tumour to be strongly positive for AE1/AE3 and CK7, whilst it was focally positive for CK20 and negative for PLAP, PSA, CEA and vimentin. Taking into account his previous urological history, the features of this tumour are consistent with metastatic TCC.

**Figure 2:**
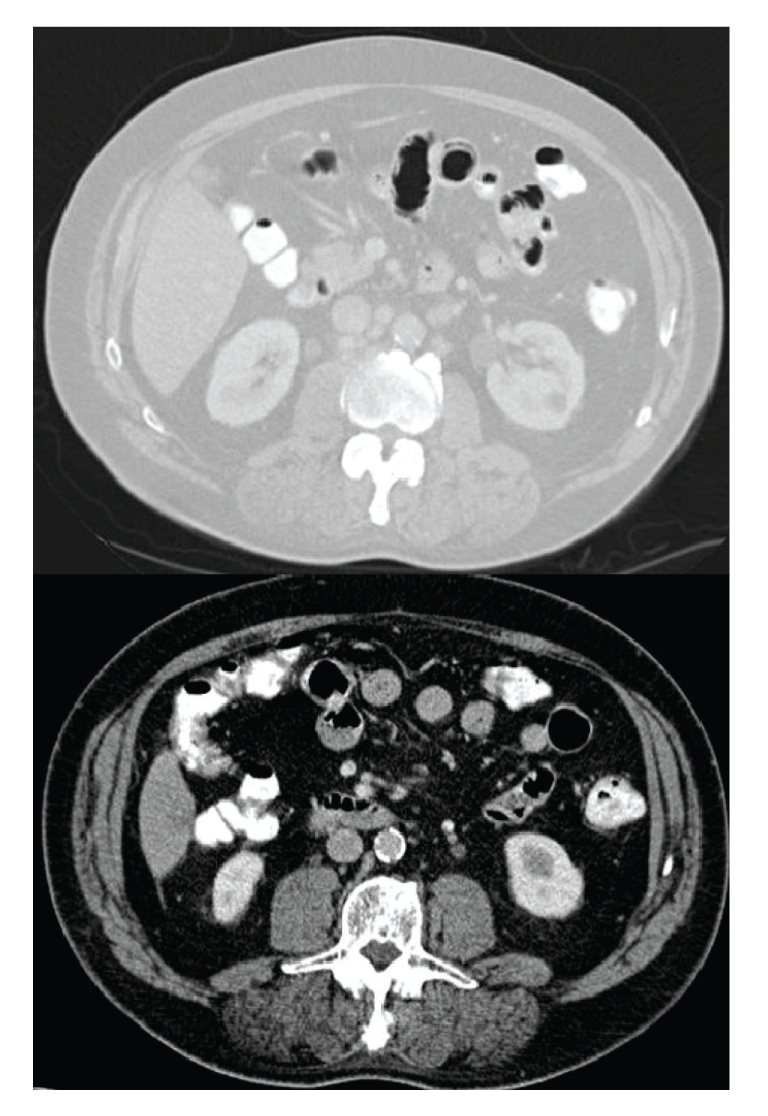
Above: CT scan June 2010 revealing abdominal para-aortic lymphadenopathy. Below: unremarkable CT scan, November 2009.

Urgent CT and bone scans were arranged (June 2010). The bone scan was unremarkable; unfortunately CT revealed abdominal para-aortic lymphadenopathy which is new since the CT in November 2009 and almost certainly due to further metastases (Figure 2: a comparison between CT scans in November 2009 and June 2010). He is now about to commence chemotherapy.

## 2. Discussion

Testicular metastasis from bladder TCC is very rare. Following a comprehensive literature search, there have been 8 reported cases from 1984 to 2006, to the best of our knowledge. Three cases have been from the UK [1–3].

Classically the mode of spread of TCC is direct, implantation, lymphatic, and haematogenous. Direct spread involves tumour growth into detrusor muscle, the ureters, prostate, urethra, uterus, vagina, perivesical fat, bowel, and pelvic side walls. Implantation may be into wounds and percutaneous catheter tracts. Lymphatic infiltration is usually to the iliac and para-aortic nodes. Haematogenous spread commonly involves the liver (38%), lung (36%), bone (27%), and adrenal glands (21%). It is important to note that haematogenous spread may involve any organ [4].

There are a number of important learning points from this case. This gentleman presented with Tis in 2002, 2005 and 2008, which seemed to respond to BCG each time it was reinstigated, and perhaps a radical cystectomy should have been performed at this point as recommended by the European Association of Urology (EAU) [5]. Also, when the patient presented in January 2010 with left testicular pain he was treated for an infection, although his symptoms improved with antibiotics, should a more open mind have been kept in view of the symptoms not resolving completely? In addition to this he was not seen again after this episode for approximately 4 months due to more than one hospital looking after him.

A key learning point is the fact that metastatic disease to the testis was never considered as a diagnosis until the histology was obtained from orchidectomy; however, this is understandable due to its rarity. Also, as this man no longer had a bladder, therefore he is unlikely to develop epididymitis secondary to urosepsis.

In conclusion a diagnosis of metastatic transitional cell carcinoma of the bladder presenting at any site must be considered in all men with bladder neoplasia.
